# Clinical implications of immune checkpoint markers and immune infiltrates in patients with thymic neuroendocrine neoplasms

**DOI:** 10.3389/fonc.2022.917743

**Published:** 2022-09-20

**Authors:** Man Liu, Wanming Hu, Yixuan Zhang, Ning Zhang, Luohai Chen, Yuan Lin, Yu Wang, Yanji Luo, Yu Guo, Minhu Chen, Jie Chen

**Affiliations:** ^1^ Department of Gastroenterology, The First Affiliated Hospital, Sun Yat-sen University, Guangzhou, China; ^2^ Department of Pathology, Sun Yat-sen Cancer Center, Guangzhou, China; ^3^ Department of Pathology, The First Affiliated Hospital, Sun Yat-sen University, Guangzhou, China; ^4^ Department of Oncology Interventional Therapy, The First Affiliated Hospital, Sun Yat-sen University, Guangzhou, China; ^5^ Department of Radiology, The First Affiliated Hospital, Sun Yat-sen University, Guangzhou, China

**Keywords:** thymic neuroendocrine neoplasms, programmed death-1, programmed death ligand-1, immune infiltrates, immune checkpoint blockade

## Abstract

The potential response of immune checkpoint blockade (ICB) in thymic neuroendocrine neoplasms (T-NEN) is largely unknown and full of great expectations. The expression of immune checkpoint molecules and immune infiltrates greatly determine the response to ICB. However, studies regarding the immune landscape in T-NEN are scarce. This work was aimed to characterize the immune landscape and its association with clinical characteristics in T-NEN. The expression of programmed cell death protein 1 (PD-1) and its ligand, programmed death ligand-1 (PD-L1), and the density of tumor-infiltrating lymphocytes (TILs), monocytes, and granulocytes were determined by immunohistochemical (IHC) staining on tumor tissues from T-NEN. Immune landscapes were delineated and correlated with clinicopathological factors. We found that T-NEN with increased immune cell infiltration and enhanced expression of PD-1/PD-L1 tended to have restricted tumor size and less metastases. A higher density of CD8^+^ TILs was associated with a significantly lower rate of bone metastasis. In addition, we presented three cases of T-NEN who progressed after multiple lines of therapies and received ICB for alternative treatment. ICB elicited durable partial responses with satisfactory safety in two patients with atypical carcinoid, but showed resistance in 1 patient with large cell neuroendocrine carcinoma. This innovative study delineated for the first time the heterogeneous immune landscape in T-NEN and identified CD8^+^ TILs as a potential marker to predict bone metastasis. An “immune-inflamed” landscape with the presence of TILs predominated in T-NEN, making T-NEN a potentially favorable target for ICB treatment. Further judicious designs of “tailor-made” clinical trials of ICB in T-NEN are urgently needed.

## Introduction

Thymic neuroendocrine neoplasms (T-NEN) are rare tumors and distinct entities of thymic malignancies. They account for 5% of all thymic tumors and less than 0.5% of all NEN, far less than the incidence of broncho-pulmonary NEN and gastroenteropancreatic (GEP) NEN (1–3). According to the most recent SEER database, the incidence of T-NEN is 0.04/100,000 per year among Asian/Pacific islanders and 0.02/100,000 per year among Caucasians (2). The rarity of T-NEN has resulted in a lack of large series and clinical trials, so there are limited guidelines or consensus statements for optimal treatment. Surgical resection is widely agreed to be the only curative method for resectable T-NEN, while chemotherapy with or without radiotherapy is recommended for patients with unresectable or metastatic disease (3). Nevertheless, the long-term outcome of patients with metastatic T-NEN remains poor and the exploration of novel treatments is urgently required in this rare tumor entity.

Cancer immunotherapy has achieved outstanding breakthroughs over the past few years, yielding pronounced clinical benefits in various tumor types (4). Immune checkpoint blockade (ICB) that targets programmed cell death protein 1 (PD-1) and its ligand, programmed death ligand-1 (PD-L1), is the most attractive immunotherapy in restoring the anti-tumor immune response. It has been approved by the Food and Drug Administration (FDA) for the treatment of various tumor types, including melanoma, non-small cell lung cancer (NSCLC), head and neck squamous cell carcinoma (HNSCC), and urothelial cancer (5). Clinical trials of ICB are currently being evaluated in NEN derived from different origins, including the gastrointestinal tract (GI), pancreas, and lung (6–9). In the phase II KEYNOTE-158 study, anti-PD-1 (pembrolizumab) showed limited antitumor activity with an objective response rate (ORR) of 3.7% in 107 patients with advanced well-differentiated neuroendocrine tumors (NET) (6). The multicohort, phase 1 KEYNOTE-028 study in patients with PD-L1-positive NET treated with pembrolizumab demonstrated an ORR of 12.0% in patients with carcinoid and 6.3% in patients with well-differentiated or moderately-differentiated pancreatic NET (7). In addition, based on a phase II basket trial of anti-CTLA-4 (ipilimumab) and anti-PD-1 (nivolumab) co-blockade, patients with high-grade neuroendocrine carcinomas (NEC) had an ORR of 44% (8/18 patients) vs. 0% in low/intermediate grade NET (0/14 patients; *p* = 0.004) (8), while among 40 NEN patients enrolled in a multiple-center phase Ib trial of anti-PD-1 (toripalimab), poorly-differentiated NEC and well-differentiated NET subgroups had similar response rates (ORR: 18.7% vs. 25.0%) (9). However, T-NEN have barely been included in previous and ongoing clinical trials due to its rarity. Therefore, no conclusions regarding the efficacy of ICB in T-NEN can be drawn yet. Nevertheless, the potential of ICB in treating T-NEN is still full of great expectations.

There is growing evidence that the expression of immune checkpoint markers and tumor-infiltrating immune cells determine the clinical response to ICB treatment (10). However, very little is known regarding the tumor microenvironment or expression of immune checkpoint molecules in T-NEN. Therefore, the aim of this study was to characterize the immune landscape in T-NEN with regard to the PD-1/PD-L1 pathway, tumor-infiltrating lymphocytes (TILs) [including helper T (Th) and cytotoxic T lymphocytes (CTLs)], and tumor-infiltrating myeloid cells (including monocytic and granulocytic cells). Furthermore, we analyzed the correlation between immunological variables and clinicopathologic parameters. We also presented clinical cases of metastatic T-NEN treated with ICB. Our study provided the first delineation of the immune landscape in T-NEN and offered clinical practice experience of ICB in patients with T-NEN, which may pave the way for a rational design of prospective clinical trials in T-NEN.

## Materials and methods

### Patient selection

This study cohort included 51 patients with histologically confirmed T-NEN (36 cases from the First Affiliated Hospital, Sun Yat-sen University, and 15 cases from Sun Yat-sen Cancer Center) from 2014 to 2021. Formalin-fixed paraffin-embedded (FFPE) tissue slides were acquired from 43 surgically resected T-NEN and 8 biopsy tissues. Data of clinical parameters were extracted from electronic patient medical records. Pathologists reviewed the tumor pathology of each case according to the 5^th^ edition of the World Health Organization (WHO) Classification of Thoracic tumors (1). T-NEN were reclassified into four categories: typical carcinoids (TC), atypical carcinoids (ATC), small cell neuroendocrine carcinomas (SCNEC), and large cell neuroendocrine carcinomas (LCNEC). The tumor-node-metastasis (TNM) stage was classified according to the 8^th^ edition AJCC Cancer Staging Manual (11). The Internal Review Board and the Ethical Review Committee of Sun Yat-sen University approved the study’s protocol. Consent was obtained from each patient after a full explanation of the purpose and nature of all procedures had been provided to the patient.

### Immunohistochemistry and image analysis

Immunohistochemistry (IHC) was performed with antibodies against PD-L1 (clone EPR19759, dilution 1:100; ab213524, Abcam) and PD-1 (clone OTI3C6, dilution 1:100; TA806807, ZSGB-BIO) to evaluate the expression of immune checkpoint molecules. IHC was performed with antibodies against CD4 (clone EP204, dilution 1:100; ZA-0519, ZSGB-BIO), CD8 (clone OTI3H6, dilution 1:100; TA802079, ZSGB-BIO), CD14 (clone SP192, dilution 1:100; ab183322, Abcam), and CD15 (clone EPR9521, dilution 1:100; ab172729, Abcam) to evaluate the level of immune infiltration. IHC was performed manually according to the following standardized procedures by experienced technicians. Four µm thick unstained slides obtained from FFPE tissue were deparaffinized in xylene and rehydrated in graded alcohols. Endogenous peroxidase activity was blocked by incubating the slides with 3% hydrogen peroxide for 10 min at room temperature, followed by incubation with antigen retrieval buffer for 20 min. Sections were transferred to PBS and incubated with the indicated antibodies overnight. Thereafter, sections were rinsed with PBS and incubated with biotinylated secondary antibody (Dako, K5007) for 20 min at room temperature. Sections were then incubated with enzyme conjugate for 10 min, followed by DAB chromogen incubation for 3–10 min (DAB detection kit, PV-6000-D, ZSGB-BIO). Lastly, sections were rinsed well with distilled water, counterstained in hematoxylin, and mounted.

Slides were scanned at ×20 magnification by the Axio Scan Z1 Slide Scanner (Zeiss, Oberkochen, Germany). Images were captured from five 1-mm^2^ areas on each slide. Quantification of positively stained cells was performed using ImagePro Plus software (Media Cybernetics, Rockville, MD, USA). The average number of cells of interest (number/mm^2^) was calculated based on the scores from the five 1-mm^2^ areas (**Supplementary Figure 1**). The tumor area containing the highest density of associated markers was designated the “hotspot”.

PD-L1 expression was evaluated both on tumor cells (TC) for the tumor proportion score (TPS) and inflammatory cells (IC) for the immune proportion score (IPS) (12). The TPS was quantified by evaluating the ratio of PD-L1-positive tumor cells to the number of all viable tumor cells. The IPS was quantified by evaluating the ratio of PD-L1-positive immune cells to the number of all infiltrated immune cells. The TPS and IPS were quantified by a professional pathologist. Following the standard recommendation by previous publications, PD-L1 expression was determined by the TPS and classified into TPS <1% (negative staining), TPS 1 to 49% (moderate staining) and TPS ≥50% (strong staining) (13, 14), or determined by the IPS and classified into IPS <1% (negative staining), IPS 1 to 9% (moderate staining) and IPS≥10% (strong staining) (14). Immune infiltrating cells with positive staining of individual markers including PD-1, CD4, CD8, CD14, and CD15 were counted in five 1-mm^2^ squares and their average number were scored into four categories: no infiltration (0 IHC^+^ cells/mm^2^), low infiltration (1~499 IHC^+^ cells/mm^2^), intermediate infiltration (500~999 IHC^+^ cells/mm^2^), and high infiltration (≥1000 IHC^+^ cells/mm^2^) (15, 16).

### Classification of tumor immune landscape

Teng et al. proposed that four different types of immune landscapes exist based on the presence or absence of TILs and PD-L1 expression (17). This stratification provides rationality for predicting the response of heterogeneous tumors with distinct immune landscapes to immunotherapy. Type I is PD-L1 positive with the presence of TILs driving the adaptive immune resistance. This type is most likely to benefit from ICB as these tumors have pre-existing TILs that are turned-off by PD-L1 engagement. Type II is PD-L1 negative without TILs, and type III is PD-L1 positive without TILs. Both types II and III would most likely not be responsive given the lack of pre-existing CTL infiltrates. Type IV is PD-L1 negative with TILs, which may still respond to ICB.

### Statistical methods

Data were analyzed using GraphPad Prism version 8 software (GraphPad, Inc., La Jolla, CA, USA). Fisher’s exact test was used to detect differences in categorical variables between groups of patients. The difference of continuous variables between two groups was evaluated by an unpaired *t*-test or unpaired *t*-test with Welch’s correction for unequal variances. All tests were two-sided, and statistical significance was declared at *p* < 0.05.

## Results

### Clinical features of T-NEN cases

**Table 1** summarizes the clinical characteristics of the cohort. The median age was 44.0 years at initial diagnosis (range: 7−76 years). The male-to-female ratio was 4.7 (42/9). Fifteen patients (15/51, 29.4%) were asymptomatic, while most patients (36/51, 70.6%) presented with varied symptoms. Seven patients (7/51,13.7%) had functional tumors. Six patients presented with Cushing syndrome and one patient with carcinoid syndrome. The other symptomatic patients had non-functional tumor-mass effects. Chest pain was the most common symptom (10/51, 19.6%). Other common symptoms were chest tightness and shortness of breath (8/51, 15.7%). Seven patients (7/51, 13.7%) complained of body pains in other sites (shoulder, back, waist, and limbs), and five patients (5/51, 9.8%) complained of cough. Four (4/51, 7.8%) cases were associated with multiple endocrine neoplasia-1 (MEN-1) with concomitant lesions in the pancreas, parathyroid gland, or pituitary.

**Table 1:** Clinical characteristics of the patient cohort.

Characteristics	Number	Frequency
***Median age (range), years***		
Initial NEN diagnosis: 44 (7 - 76)		
***Gender***		
- Male	42	82.40%
- Female	9	17.60%
***Symptoms***		
- Chest tightness and shortness of breath	8	15.70%
- Chest pain	10	19.60%
- Pain in the other sites (eg. Shoulder, back, waist, limbs, *etc* )	7	13.70%
- Cough	5	9.80%
- Cushing syndromes (eg. Central obesity,	6	11.80%
- polytrichia, hypertension, hypokalemia, *etc*)		
- Carcinoid syndromes (eg. Flushing, palpitation)	1	2.00%
None	15	29.40%
***Tumor size, cm***		
Median (range): 7.0 (1.2-19.0)		
***Functionality***		
- No	44	86.30%
- Yes	7	13.70%
***MEN-1***		
- No	47	92.20%
- Yes	4	7.80%
***Histology***		
Typical carcinoid	0	0%
Atypical carcinoid	44	86.30%
Neuroendocrine carcinoma	7	13.70%
Large-cell	3	5.90%
Small-cell	4	7.80%
**AJCC staging**		
- I	4	7.80%
- II	1	2.00%
- IIIA	4	7.80%
- IIIB	7	13.70%
- IVA	9	17.60%
- IVB	26	51.00%
***Metastases***		
- Lymph nodes	28	54.90%
- Bone	16	31.40%
- Lung	8	15.70%
- Pancreas	4	7.80%
- Liver	2	3.90%
- Brain	2	3.90%
- Breast	2	3.90%
- Adrenal gland	3	5.90%
- Orbits	1	2.00%
- None	15	29.40%

On histological examination, the majority (44/51, 86.3%) were ATC. Only seven cases (7/51, 13.7%) were NEC, among whom four were SCNEC and three were LCNEC. No TC was included. Most of the T-NEN were diagnosed at advanced stages, including 26 (26/51, 51.0%) stage IVB patients, nine (9/51, 17.6%) stage IVA patients, seven (7/51, 13.7%) stage IIIB patients, four (4/51, 7.8%) stage IIIA patients, one (1/51, 2.0%) stage II patients, and four (4/51, 7.8%) stage I patients. Unlike GEP-NEN, which often develop liver metastases, T-NEN in this cohort mostly metastasized to lymph nodes (28/51, 54.9%), bone (16/51, 31.4%), and lung (8/51, 15.7%). Four cases (4/51, 7.8%) developed pancreas metastases, two cases (2/51, 3.9%) developed liver metastases, two cases (2/51, 3.9%) developed brain metastases, two cases (2/51, 3.9%) developed breast metastases, and three cases (3/51, 5.9%) developed adrenal gland metastases. A rare metastatic site in the orbit was also identified in one case (1/51, 2.0%). Fifteen patients (15/51, 29.4%) had no metastatic disease until the last follow-up.

### Immune checkpoint markers PD-L1 and PD-1 expression patterns in T-NEN

**Figure 1:**
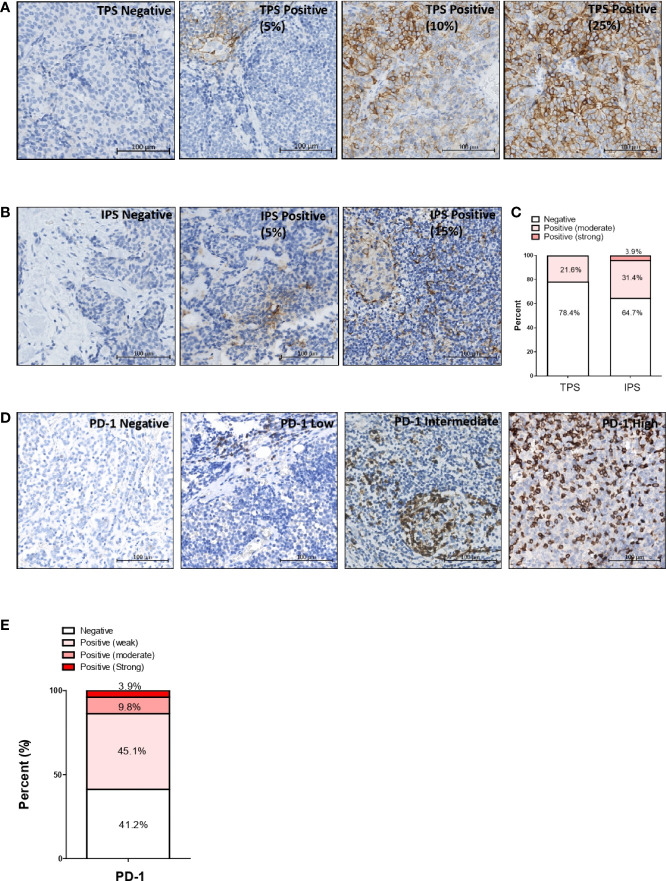
Expression pattern of immune checkpoint markers PD-L1 and PD-1 in T-NEN. Representative PD-L1 IHC staining images of T-NEN are showed. **(A)** Different levels of PD-L1 staining (brown) on tumor cells are showed. **(B)** Different levels of staining (brown) on immune cells are showed. **(C)** Proportion of stratified PD-L1 intensity according to TPS and IPS evaluation is displayed. **(D)** Different infiltration levels of PD-1-positive cells are shown with representative IHC staining images. **(E)** Proportion of stratified PD-1 intensity in T-NEN is displayed. Remark: Representative IHC staining images are shown in an area of 0.09 mm^2^ (0.3 mm×0.3 mm) captured from each slide.

Positive expression of PD-L1 was observed in 20 cases (20/51, 39.2%), including two cases (2/51, 3.9%) with expression exclusively on tumor cells, nine cases (9/51, 17.6%) with expression exclusively on immune cells, and nine cases (9/51, 17.6%) with expression on both tumor cells and immune cells. All TPS-positive tumors had moderate staining of PD-L1, with TPS ranging from 1% to 25%. In IPS-positive tumors, the PD-L1 staining intensity was moderate in 16 (16/51, 31.4%) cases and strong in two (2/51, 3.9%) cases. Representative PD-L1 IHC staining images in T-NEN are shown in **Figure 1A** (TPS) and **1B** (IPS). The proportion of stratified PD-L1 intensity according to TPS and IPS evaluations was calculated and is displayed in **Figure 1C**.

Intra-tumoral infiltration of PD-1-positive immune cells was observed in 30 samples (30/51, 58.8%). The average infiltrating level was low, intermediate, and high in 23 (23/51, 45.1%), five (5/51, 9.8%), and two (2/51, 3.9%) samples, respectively. Representative PD-1 IHC staining images in T-NEN are shown in **Figure 1D**. The proportion of stratified infiltration levels of PD-1^+^ cells was calculated and is displayed in **Figure 1E**.

### Immune cell infiltration in T-NEN and classification of the tumor immune landscape based on TILs and PD-L1

**Figure 2:**
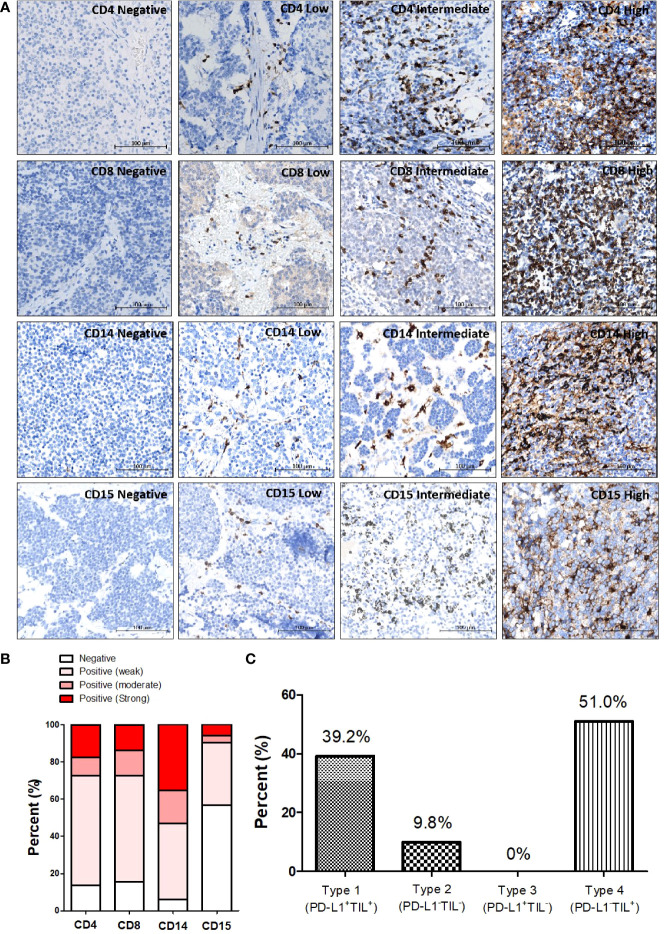
Patterns of different immune cell infiltrations in T-NEN. **(A)** Different infiltration levels of CD4^+^ TILs, CD8^+^ TILs, CD14^+^ monocytes, and CD15^+^ granulocytes in T-NEN are shown with representative IHC-stained images. **(B)** Proportion of stratified immune cell infiltration levels in T-NEN is displayed. **(C)** Classification of the immune landscape according to the PD-L1 overall/TILs pattern in T-NEN.

T-NEN tissues were evaluated with CD4 and CD8 staining for lymphocyte infiltration and with CD14 and CD15 staining for myeloid cell infiltration. Representative CD4, CD8, CD14, and CD15 IHC staining images with different intensities in T-NEN are shown in **Figure 2A**. The proportion of stratified infiltration levels of each cell type was calculated and is displayed in **Figure 2B**. CD4^+^ Th cell infiltration was observed in 44 patients (44/51, 86.3%) with nine patients (9/51, 17.6%) showing high infiltration. CD8^+^ CTL infiltration was observed in 43 patients (43/51, 84.3%) with seven patients (7/51, 13.7%) showing high infiltration. CD14^+^ monocyte infiltration was observed in 48 patients (48/51, 94.1%) with 18 patients (18/51, 35.3%) showing high infiltration. CD15^+^ granulocyte infiltration was observed in 22 patients (22/51, 43.1%) with three patients (3/51, 5.9%) showing high infiltration.

In this T-NEN cohort, 20 patients (20/51, 39.2%) presented with a type I landscape (PD-L1^+^TILs^+^) and five patients (5/51, 9.8%) with a type II landscape (PD-L1^-^TILs^-^). No patients presented with a type III landscape (PD-L1^+^TILs^-^), and the majority of the T-NEN (26/51, 51%) displayed a type IV landscape (PD-L1^-^TILs^+^). The percent of each category of tumor immune landscape is displayed in **Figure 2C**.

### Comparison analysis of the immune landscape between well-differentiated ATC and poorly-differentiated NEC

Because well-differentiated ATC and poorly-differentiated NEC harbor distinctly different biological and molecular characteristics, we compared their immune landscapes (**Table 2**). The analysis showed significantly higher CD4^+^ lymphocyte infiltrates in ATC compared to NEC (CD4^+^ hotspot score: 1,277 ± 297 cells/mm^2^ vs. 387 ± 143 cells/mm^2^, *p* = 0.0097; CD4^+^ average score: 510 ± 111 vs.141 ± 55, *p* = 0.0046). It was also notable that PD-1 expression was significantly higher in ATC compared to NEC (PD-1^+^ hotspot score: 487 ± 144 cells/mm^2^ vs. 14 ± 7 cells/mm^2^, *p* = 0.0020; PD-1^+^ average score: 199 ± 60 cells/mm^2^ vs. 3 ± 1 cells/mm^2^, *p* = 0.0021).

**Table 2:** Comparison analysis of immune landscape between well-differentiated ATC and poorly-differentiated NEC.

Parameters	ATC (n = 44)	NEC (n = 7)	*P* value
**CD4^+^ cells intensity (cells/mm^2^)**			
-Hotspot	1277±297	387±143	0.0097**
-Average	510±111	141±55	0.0046**
**CD8^+^ cells intensity (cells/mm^2^)**			
-Hotspot	1171±314	767±315	0.4
-Average	483±161	380±177	0.7
**CD14^+^ cells intensity (cells/mm^2^)**			
-Hotspot	1771±283	2153±651	0.6
-Average	850±133	1229±405	0.4
**CD15^+^ cells intensity (cells/mm^2^)**			
-Hotspot	501±216	251±192	0.4
-Average	235±92	72±55	0.1
**PD-1^+^ cells intensity (cells/mm^2^)**			
-Hotspot	487±144	14±7	0.0020**
-Average	199±60	3±1	0.0021**
**PD-L1 TPS, n (%)**			
-Negative	35 (79.5%)	5 (71.4%)	0.6
-Positive	9 (20.5%)	2 (28.6%)	
**PD-L1 IPS, n (%)**			
-Negative	29 (65.9%)	4 (57.1%)	0.7
-Positive	15 (34.1%)	3 (42.9%)	
**Immune landscape**			
-Type 1 (PD-L1^+^TIL^+^)	16 (36.4%)	4 (57.1%)	0.5^a^ 0.7^b^ 1.0^c^
-Type 2 (PD-L1^-^TIL^-^)	5 (11.4%)	0 (0.0%)	
-Type 4 (PD-L1^-^TIL^+^)	23 (52.2%)	3 (42.9%)	

Remark: Data of continuous variables are presented as mean ± SEM, the differences between two groups were evaluated by unpaired T test, or unpaired T test with Welch's correction for unequal variances; data of categorical variables are presented as case numbers and calculated frequencies, the differences were evaluated by Fisher’s exact test. P value < 0.01 is marked with **. The bold values indicate significant values. a: type 1 vs type 2; b: type 1 vs type 3; c: type 2 vs type 3.

### Correlation analysis between immune phenotype and tumor size in T-NEN

We analyzed the correlation between immune phenotype and tumor size in T-NEN (**Table 3**). We stratified T-NEN into tumors less than a median size (7 cm) and tumors greater than or equal to a median size (7 cm) and compared their differences in terms of immune phenotype (**Table 3**). Compared to tumors less than the median size, tumors greater than or equal to the median size tended to have less CD4^+^ and CD8^+^ lymphocyte infiltration, lower PD-1 expression, and less CD15^+^ granulocytic cell infiltration. However, their differences did not reach statistical significance. CD14^+^ monocytic cells differed significantly among the groups, showing that tumors less than the median size had significantly higher CD14^+^ monocytic cell infiltration than tumors greater than or equal to the median size (hotspot: 2443 ± 393 cells/mm^2^ vs. 1273 ± 311 cells/mm^2^, *p* = 0.02). From the perspective of the immune landscape, T-NEN with a type II landscape (PD-L1^-^TILs^-^), which is termed the “immunologically ignorant” phenotype, grew larger since T-NEN with a type II landscape all fell in a group of tumors greater than or equal to the median size (7 cm). The above results suggested that the “immunologically cold” T-NEN may grow larger than the “immunologically hot” T-NEN.

**Table 3:** Correlation analysis of immune phenotype and tumor size in T-NEN .

Parameters	Tumor size < median size(n = 24)	Tumor size ≥ median size (n = 27)	*P* value
**CD4^+^ cells intensity (cells/mm^2^)**			
-Hotspot	1557±454	798±271	0.2
-Average	666±177	276±85	0.1
**CD8^+^ cells intensity (cells/mm^2^)**			
-Hotspot	1761±540	665±166	0.1
-Average	717±284	288±72	0.2
**CD14^+^ cells intensity (cells/mm^2^)**			
-Hotspot	2443±393	1273±311	0.02*
-Average	1173±189	662±161	0.04*
**CD15^+^ cells intensity (cells/mm^2^)**			
-Hotspot	728±385	235±88	0.2
-Average	345±162	96±39	0.1
**PD-1^+^ cells intensity (cells/mm^2^)**			
-Hotspot	572±200	289±157	0.3
-Average	257±94	96±50	0.1
**PD-L1 TPS, n (%)**			
-Negative	19 (79.2%)	21 (77.8%)	1.0
-Positive	5 (20.8%)	6 (22.2%)	
**PD-L1 IPS, n (%)**			
-Negative	16 (66.7%)	17 (63.0%)	1.0
-Positive	8 (33.3%)	10 (37%)	
**Immune landscape**			
-Type 1 (PD-L1^+^TIL^+^)	9 (37.5%)	11 (40.7%)	0.1^a^ 0.6^b^ 0.04^c^*
-Type 2 (PD-L1^-^TIL^-^)	0 (0.0%)	5 (18.6%)	
-Type 4 (PD-L1^-^TIL^+^)	15 (62.5%)	11 (40.7%)	

Remark: Data of continuous variables are presented as mean ± SEM, the differences between two groups were evaluated by unpaired T test, or unpaired T test with welch's correction for unequal variances; data of categorical variables are presented as case number and calculated frequency, the differences were evaluated by Fisher’s exact test. P value < 0.05 is significant and marked with *. The bold values indicate significant values. a: type 1 vs type 2; b: type 1 vs type 3; c: type 2 vs type 3.

### Correlation between immune phenotype and metastasized diseases in T-NEN

**Figure 3:**
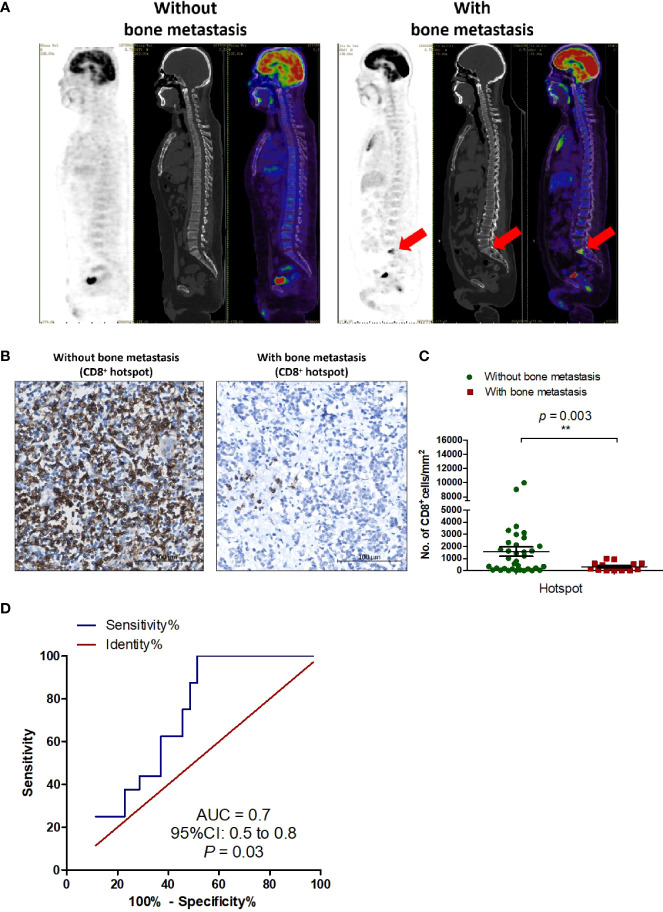
CD8^+^ TILs are a potential immunological marker for predicting bone metastasis in T-NEN. **(A)** Representative ^18^F-FDG PET/CT scan imaging of T-NEN patients with or without bone metastasis. **(B)** Representative IHC images showing CD8^+^ TILs deposition in T-NEN with and without bone metastasis. **(C)** Level of CD8^+^ TILs (hotspot of CD8^+^ cells/mm^2^) was quantified in each case. Scatter plots of CD8^+^ hotspot scores in patients with bone metastasis (n = 16) and without bone metastasis (n = 35) were displayed and comparison analysis was performed using the unpaired *t*-test. Data represent the mean ± SEM, ^**^ *p* < 0.01. **(D)** ROC was plotted to assess the predictive capacity of CD8^+^ TILs to predict bone metastasis.

Because metastasis is a critical biological characteristic of tumors and is associated with a worse outcome, we analyzed the correlation between immune phenotype and metastasis in T-NEN (**Table 4**). The analysis showed that none of the clinicopathologic parameters (age, gender, symptomatic, tumor size, pathology) differed significantly between the metastatic and non-metastatic groups. We next evaluated the association between immune phenotype and different sites of metastases. The analysis showed significantly higher CD8^+^ scores in patients without bone metastasis compared to patients with bone metastasis (hotspot score: 1573 ± 386 cells/mm^2^ vs. 323 ± 82 cells/mm^2^, *p* = 0.003; average score: 640 ± 200 cells/mm^2^ vs. 161 ± 47 cells/mm^2^, *p* = 0.025) (**Figures 3A–C**; **Table 4**), suggesting a potential role of CD8^+^ T cells in suppressing bone metastasis. We also analyzed the correlation between CD8^+^ TILs and metastases to other sites such as lymph nodes and lung, but no significant correlation could be identified (**Table 4**).

**Table 4:** Patient clinicopathological and immunological characteristics and their correlations with metastasized disease.

	Parameters	Lymph node metastasis		Bone metastasis				Lung metastasis			Overall metastasis		
Parameters	No(n = 23)	Yes(n = 28)	*P*	No(n = 35)	Yes(n = 16)	*P*	No(n = 43)	Yes(n = 8)	*P*	No(n = 15)	Yes(n = 36)	*P*
**Age (year)**	43.0±2.6	42.7±2.2	0.9	43.4±2.2	41.5±2.2	0.6	43.2±1.7	40.8±5.4	0.6	44.5±3.7	42.1±1.8	0.5
**Gender, n (%)**												
-Female	3 (13.0%)	6 (21.4%)	0.5	4 (11.4%)	5 (31.3%)	0.1	7 (16.3%)	2 (25%)	0.6	2 (13.3%)	7 (19.4%)	0.7
-Male	20 (87.0%)	22 (78.6%)		31 (88.6%)	11 (68.7%)		36 (83.7%)	6 (75%)		13 (86.7%)	29 (80.6%)	
**Symptomatic, n (%)**												
-No	10 (43.5%)	5 (17.9%)	0.1	13 (37.1%)	2 (12.5%)	0.1	13 (30.2%)	2 (25%)	1.0	6 (40%)	9 (25%)	0.3
-Yes	13 (56.5%)	23 (82.1%)		22 (62.9%)	14 (87.5%)		30 (69.8%)	6 (75%)		9 (60%)	27 (75%)	
**Tumor size^2^ (cm)**	8.1±1.0	7.2±0.7	0.4	7.1±0.7	8.7±1.1	0.2	7.4±0.6	8.4±1.7	0.5	6.7±0.9	8.0±0.7	0.3
**Pathology, n (%)**												
-Atypical carcinoid	22 (95.7%)	22 (78.6%)	0.1	30 (85.7%)	14 (87.5%)	1.0	37 (86.0%)	7 (87.5%)	1.0	15 (100%)	29 (80.6%)	0.1
-NEC	1 (4.3%)	6 (21.4%)		5 (14.3%)	2 (12.5%)		6 (14.0%)	1 (12.5%)		0 (0%)	7 (19.4%)	
**CD4^+^ cells intensity (cells/mm^2^)**												
-Hotspot	1354±491	991±255	0.5	1324±355	786±285	0.2	1259±302	593±308	0.1	1899±715.	845±207	0.2
-Average	453±131	464±143	0.9	517±130	333±127	0.4	482±111	340±180	0.6	626±185	390±114	0.3
**CD8^+^ cells intensity (cells/mm^2^)**												
-Hotspot	1329±456	1059±344	0.6	1573±386	323±82	**0.003** **	1305±322	515±256	0.1	1796±664	924±275	0.2
-Average	406±111	560±241	0.6	640±200	161±47	**0.025** *	533±165	260±119	0.2	522±154	477±190	0.9
**CD14^+^ cells intensity (cells/mm^2^)**												
-Hotspot	1546±357	2052±369	0.3	2066±327	1293±391	0.2	1947±299	1163±301	0.1	1416±429	1993±319	0.3
-Average	729±154	981±195	0.3	1010±159	665±201	0.2	967±146	552±142	0.1	643±157	1010±165	0.2
**CD15^+^ cells intensity (cells/mm^2^)**												
-Hotspot	614±397	347±114	0.5	499±258	397±213	0.8	522±221	170±170	0.2	732±579	357±121	0.5
-Average	260±153	174±77	0.6	214±103	210±125	1.0	234±93	99±99	0.3	272±203	188±78	0.7
**PD-1^+^ cells intensity (cells/mm^2^)**												
-Hotspot	454±210	396±154	0.8	509±174	231±121	0.2	450±144	272±212	0.6	687±309	311±122	0.3
-Average	159±70	182±77	0.8	204±71	102±62	0.3	178±59	141±118	0.8	240±103	143±61	0.4
**PD-L1 TPS, n (%)**												
-Negative	15 (37.5%)	25 (62.5%)	**0.04** *	25 (62.5%)	15 (37.5%)	0.1	33 (82.5%)	7 (17.5%)	0.7	9 (22.5%)	31 (77.5%)	0.1
-Positive	8 (72.7%)	3 (27.3%)		10 (90.9%)	1 (9.1%)		10 (90.9%)	1 (9.1%)		6 (54.5%)	5 (45.5%)	
**PD-L1 IPS**												
-Negative	11 (33.3%)	22 (66.7%)	**0.03** *	21 (63.6%)	12 (36.4%)	0.4	27 (81.8%)	6 (18.2%)	0.7	5 (15.2%)	28 (84.8%)	**0.004** **
-Positive	12 (66.7%)	6 (33.3%)		14 (77.8%)	4 (22.2%)		16 (88.9%)	2 (11.1%)		10 (55.6%)	8 (44.4%)	
**Immune landscape**												
-Type 1 (PD-L1^+^TIL^+^)	13 (65.0%)	7 (35.0%)	0.4^a^ **0.03^b^** * 1.0^c^	16 (80.0%)	4 (20.0%)	0.6^a^ 0.2^b^ 1.0^c^	18 (90.0%)	2 (10.0%)	0.5^a^ 0.4^b^ 1.0^c^	10 (50.0%)	10 (50.0%)	0.3^a^ **0.02^b^** * 1.0^c^
-Type 2 (PD-L1^-^TIL^-^)	2 (40.0%)	3 (60.0%)		3 (60.0%)	2 (40.0%)		4 (80.0%)	1 (20.0%)		1 (20.0%)	4 (80.0%)	
-Type 4 (PD-L1^-^TIL^+^)	8 (30.8%)	18 (69.2%)		16 (61.5%)	10 (38.5%)		21 (80.8%)	5 (19.2%)		4 (15.4%)	22 (84.6%)	

Remark: Data of continuous variables are presented as mean ± SEM, the differences between two groups were evaluated by unpaired T test, or unpaired T test with welch's correction for unequal variances; data of categorical variables are presented as case number and calculated frequency, the differences were evaluated by Fisher’s exact test. P value < 0.05 is significant and marked with * and p value < 0.01 is marked with **. The bold values indicate significant values. a: type 1 vs type 2; b: type 1 vs type 3; c: type 2 vs type 3.

ROC curve analysis was performed (**Figure 3D**) to identify the optimal cut-off number of CD8^+^ TILs capable of predicting bone metastasis. In this T-NEN cohort, a CD8^+^ TILs hotspot score greater than 534 cells/mm^2^ distinguished between patients with and without bone metastases with a sensitivity of 48.6%, specificity of 100%, and an area under the curve (AUC) of 0.7 (95% CI = 0.5–0.8, *p* = 0.03). Patients with a CD8^+^ TILs hotspot score greater than 534 cells/mm^2^ had significantly lower rates of bone metastases than patients with CD8^+^ TILs hotspot score less than or equal to 534 cells/mm^2^ (16.7% vs. 44.4%, *p* = 0.04). Therefore, CD8^+^ TILs served as a valuable prediction marker with high specificity for bone metastasis in patients with T-NEN.

Applying positive or negative PD-L1 expression as a dichotomous variable, a significant association was found between lymph node metastases and PD-L1 presence. Lymph node metastasis rates were significantly higher in PD-L1 negative cases compared to positive cases (TPS: 62.5% vs. 27.3%, *p* = 0.04; IPS: 66.7% vs. 33.3%, *p* = 0.03). Besides, the overall metastasis rate was also significantly higher in PD-L1 IPS negative cases than positive cases (84.8% vs. 44.4%, *p* = 0.004). When analyzing metastasis rates in different immune landscape categories, we found significantly higher lymph node or overall metastasis rates in type IV (PD-L1^-^TIL^+^) cases than type I (PD-L1^+^TIL^+^) cases (lymph node metastasis: 69.2% vs. 35.0%, *p* = 0.03; overall metastasis: 84.6% vs. 50.0%, *p* = 0.02), suggesting a negative correlation between PD-L1 presence and metastatic status in TIL^+^ tumors.

### Clinical practice of ICB with toripalimab in patients with metastatic T-NEN

**Figure 4:**
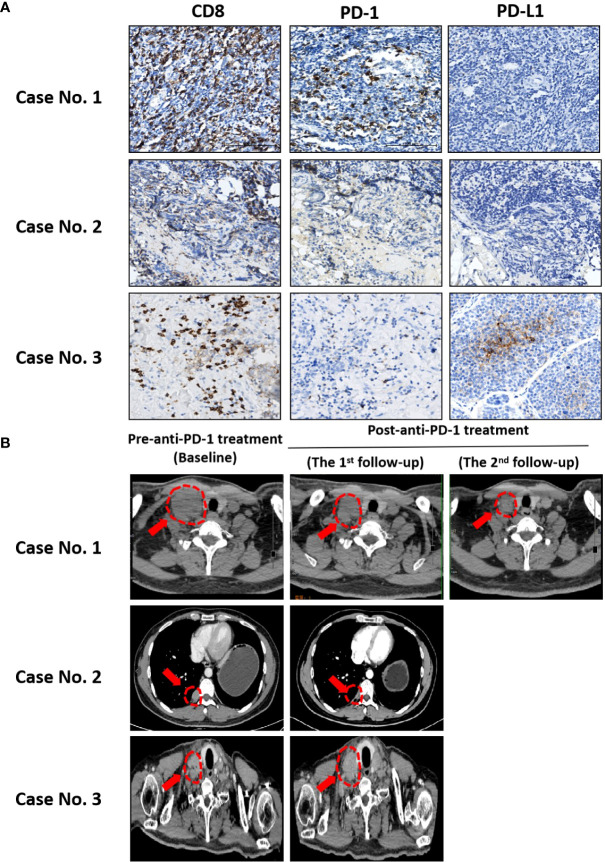
Clinical practice of ICB with toripalimab in patients with metastatic T-NEN. **(A)** Representative IHC images showing CD8, PD-1, and PD-L1 expression patterns in case No.1, 2, and 3. **(B)** CT scans at baseline (pre-anti-PD-1 treatment) and re-examinations after anti-PD-1 treatment at indicated time points are shown. The arrow points to the lesions (with red marks) that changed significantly upon toripalimab treatment.

Recent evidence has pointed out that CD8^+^ TILs were the most powerful effector immune cells in eliciting an anti-tumor immune response and form the backbone of successful immunotherapy (18). Therefore, we tested the efficacy of ICB with toripalimab (Shanghai Junshi Bioscience Co., Ltd in China) (9), a PD-1 monoclonal antibody, in three T-NEN patients with high levels of CD8^+^ TILs infiltration. The characteristics of the patients are listed in **Table 5**. The expression patterns of CD8, PD-1, and PD-L1 are shown in **Figure 4A**. All three patients progressed following prior treatments, including surgery, radiotherapy, and chemotherapy, but had no satisfactory alternative treatment options. Considering the presence of high CD8^+^ TILs infiltration, we treated the three patients with toripalimab, 240 mg every three weeks after obtaining the patients’ explicit and informed consents.

**Table 5:** Characteristics of the patients received ICB treatment.

Case No.	Age	Gender	Pathology	Mitosis (2mm^2^)	Primary tumor size(mm×mm)	Metastatic sites	CD8^+^ TILs	PD-1	PD-L1	ICB cycles	Response to ICB
1	24	Male	ATC	8	55 ×50	Lymphnodes	9054/mm^2^ (high)	1637/mm^2^(high)	TPS: 0%IPS: 0%	24	PR
2	35	Male	ATC	5	43×32	Lymphnodes	1623/mm2 (high)	466/mm2(moderate)	TPS: 0%IPS: 0%	13	PR
3	61	Male	LCNEC	15	49×55	Lymphnodes	3325/mm2 (high)	0/mm2(absence)	TPS: 5%IPS: 0%	4	PD

Patient No.1 is a case of thymic ATC diagnosed in April 2014, and underwent surgery to remove mediastinal mass and adjacent lymph nodes, followed by 4 cycles of chemotherapy with EP (Etoposide plus Cisplatin) regimen, 28 cycles of chest radiotherapy and 12 cycles of CAPTEM (Capecitabine plus Temozolomide). However, multiple enlarged lymph node metastases still existed, especially a palpable mass on the right side of the neck was noted. No partial response (PR) could be achieved upon above treatments. Since a high CD8^+^ TILs infiltration (hotspot: 9054/mm^2^) was present in this patient’s T-NEN specimen, immunotherapy with toripalimab was initiated since December 2020. A satisfactory PR was achieved, with a significant shrinkage of the palpable mass on the right side of the neck from 48mm×40mm to 26mm×13mm at September 2021 (**Figure 4B**). The patient had received toripalimab for 18 months and kept stable disease (SD) until the last follow-up date (July 2022). This patient is alive and has survived for 99 months since initial diagnosis.

Patient No.2 is a case of thymic ATC diagnosed in October 2017, and underwent surgery followed by radiotherapy. A recurrence in mediastinum with multiple lymph node metastases in mediastinum and mammary area was identified in August 2020. CAPTEM chemotherapy was administrated, but follow-up scan showed progressive disease (PD). Since a high CD8^+^ TILs infiltration (hotspot: 1623 cells/mm^2^) was present in this patient’s T-NEN specimen, immunotherapy with toripalimab treatment was initiated since May 2021. A PR was achieved, with a significant shrinkage of the metastatic lymph node (**Figure 4B**) after 4 cycles of toripalimab treatment. The patient kept SD for 10 months upon toripalimab treatment, but progressed in March 2022. Toripalimab treatment was stopped and changed to EP regimen since then. This patient is alive and has survived for 57 months since initial diagnosis.

Patient No.3 is a case of thymic LCNEC diagnosed in April 2018. Surgical resection of primary mass with lymph nodes dissection was performed in May 2018, but multiple lymph node metastases still existed at mediastinum, supraclavicular fossa, superior phrenic, retroperitoneal, *etc.* The patient was treated with chemotherapy with EP regimen since September 2019 and achieved SD for months, but progressed at September 2020. EP regimen was stopped and changed to CAPTEM regimen, but the disease still progressed in March 2021. Since a high CD8^+^ TILs infiltration (hotspot: 3325 cells/mm^2^) was present in this patient’s T-NEN specimen, immunotherapy with toripalimab was initiated since April 2021. However, follow-up CT scan in August 2021 showed PD (**Figure 4B**), therefore the toripalimab treatment stopped since then. Without satisfactory alterative treatment options, the disease progressed and the patient died in January 2022. The survival duration of this case is 44 months since initial diagnosis.

During toripalimab treatment, immune-related adverse events (irAE) including dermatologic, pulmonary, cardiac, endocrine, gastrointestinal, hepatobiliary and renal toxicities, as well as other general symptoms such as fatigue and appetite loss were recorded (**Supplementary Table 1**) (19). No patients developed any Grade 3-4 irAE. Only 1 patient complained a transient fatigue and appetite loss but recovered soon without special treatment. In brief, toripalimab demonstrated a sustained antitumor activity in two patients with metastatic T-NEN and was well-tolerated.

## Discussion

Delineation of the immune checkpoint molecule PD-L1/PD-1 expression pattern and immune cell infiltration in tumors is critical for defining the immune environment status of T-NEN and identifying subgroups of patients who would potentially benefit from immunotherapy approach (20). However, due to the rarity of T-NEN, the literature regarding the descriptive immune landscape in T-NEN is scarce. To the best of our knowledge, this study represented the first series to investigate the immune landscape and its association with clinical characteristics and the potential response to immunotherapy in T-NEN.

The clinical behavior of T-NEN is highly unpredictable. Well-differentiated cases can unexpectedly be associated with widely distributed metastases and very poor prognosis (21). However, current WHO grading can only partially indicate T-NEN’s clinical behavior. Unlike GEP-NEN, which mostly develop liver metastasis, T-NEN predominantly metastasize to bones and are associated with a worse outcome (21). In this cohort, bone metastases were present in 31.4% (16/51) of T-NEN patients, which is consistent with the incidence of 33% reported in the literature (22). Currently, there is no reliable biomarker that can predict bone metastasis in T-NEN, and no underlying mechanisms have been uncovered to address the issue. In our series, a significant correlation between bone metastasis and the level of CD8^+^ TILs was reported for the first time in T-NEN, showing that higher level of CD8^+^ TILs was associated with less bone metastasis. This finding is in accordance with a previous observation in pulmonary NEN reported by Wang et al (23), which demonstrated that higher CD8^+^ T cell densities were significantly associated with the absence of vascular invasion, negative lymph node metastasis, and lower clinical staging. We further identified the optimal cut-off value for the CD8^+^ TILs hotspot score (534 cells/mm^2^) to predict bone metastasis. We showed that T-NEN with a CD8^+^ TILs hotspot score greater than the cut-off value tended to have fewer bone metastases than T-NEN with a CD8^+^ TILs hotspot score less than or equal to the cut-off value. Therefore, CD8^+^ TILs possessed a potential value for bone metastasis subgroup discrimination. The possible reason for less bone metastasis in cases with higher CD8^+^ TILs might relate to the essential role of CD8^+^ TILs in immune surveillance (24). CD8^+^ TILs act as an inspector who eliminate abnormal metastazied tumor cells as soon as they detect them, thereby blocking the distant metastasis of cancer (24). Besides, it has been validated in a mouse melanoma model showing that CD8^+^ TILs activation diminished bone metastasis while CD8^+^ TILs depletion enhanced it (25). However, the exact role of CD8^+^ TILs in T-NEN still needs our further mechanistic investigation. Besides, PD-L1 status was also correlated with metastasis in this cohort, showing that lymph node metastasis and overall metastasis rates were significantly lower in T-NEN with PD-L1 expression. This suggests that PD-L1-involved adaptive immune activation within the intratumoral compartment may prevent tumor cell dissemination. However, the detailed mechanisms require further elucidation.

PD-L1 expression on both tumor cells and immune cells, as well as PD-1 expression on tumor-infiltrating lymphocytes, has been identified as a critical factor that determines the response to immunotherapies targeting the PD-L1/PD-1 axis (26), and have demonstrated to be promising predictive and prognostic biomarkers in NEN (27). However, the status of PD-L1/PD-1 expression has not been established in T-NEN yet. The results of our analysis showed that positive expression of PD-L1 was observed in 20 cases (20/51, 39.2%). The frequency of positive PD-L1 expression in T-NEN is comparable with the frequency in 159 pulmonary NENs (72/159, 45%) reported by Wang et al (23), and is relatively higher than the frequency in 106 pancreatic NETs (26/106, 25%) reported by Mehnert et al (7), or in 57 GEP-NENs (16/57, 28%) reported by Cavalcanti et al (28). PD-L1 expression was more frequent on immune cells (18/51, 35.3%) than on tumor cells (11/51, 21.6%), thus also confirming the previous observations in GEP-NEN and pulmonary NEN (29–31). The proportion of positive PD-L1 expression was slightly higher in NEC (TPS: 28.6%; IPS: 42.9%) than in ATC (TPS: 20.5%; IPS: 34.1%) in this T-NEN cohort, which is in line with a previous finding that the more aggressive the NEN, the higher the expression of PD-L1 (28). Since lung and thymic NEN are often grouped within one unique group, and treatment strategies for thymic NEC are often extrapolated from the treatment paradigm for small-cell lung cancer (SCLC) (3), we then compared our study to previous investigations in SCLC. According to data reported by L. Bonanno et al., PD-L1 was expressed on tumor cells and tumor-infiltrating immune cells in 25% and 40% of SCLC cases, respectively, and CD8^+^ TILs were present in 59% of SCLC samples (32). Hui Yu et al. reported that the overall prevalence of PD-L1 expression was 16.5% in tumor cells and 44.8% in tumor-infiltrating immune cells in their SCLC cohort (33). In our study, PD-L1 was expressed on tumor cells in two thymic NEC cases (2/7, 28.6%) and on tumor-infiltrating immune cells in three thymic NEC cases (3/7, 42.9%), which are comparable with the frequency of PD-L1 presence in SCLC (32, 33). CD8+ TILs were present in six (6/7, 85.7%) of thymic NEC samples in our study, which is more frequent than CD8+ TILs observed in SCLC samples (32). As for PD-1, only a minority of samples (2/51, 3.9%) stained strongly positive for PD-1, suggesting that infiltrating lymphocytes lack effective priming by tumor neoantigens. This is in line with the low mutational burden of T-NEN reported in the literature (34, 35).

The immune microenvironment, which is highly heterogeneous and complex, is composed of diverse immune cells and includes both lymphoid cells and myeloid cells. The roles of lymphoid cells such as CD4^+^ TILs and CD8^+^ TILs have been well elucidated in anti-tumor responses, while the complex role of myeloid cells such as CD14^+^ monocytes and CD15^+^ granulocytes have been relatively underexplored. Other than the well-known function in responding to inflammation, infection, and injury, tumor-infiltrated monocytes and granulocytes also play fundamental roles in orchestrating the immune landscape and regulating tumor progression (36). The relevance of TILs and myeloid cells in tumor compartments for predicting immunotherapy response has been widely validated in various cancer types (20). However, information about their distribution in T-NEN is scarce. In this T-NEN cohort, we demonstrated that the majority of T-NEN harbored various degrees of CD4^+^ TILs (86.3%) and CD8^+^ TILs (84.3%), as well as CD14^+^ monocyte (94.1%) and CD15^+^ granulocyte (43.1%) infiltration, thus displaying an “immune-inflamed” landscape and making immunotherapy a rational way to tackle tumor progression in T-NEN.

According to studies in pulmonary NEN and GEP-NEN, poorly-differentiated NEC are more frequently associated with high levels of microsatellite instability (MSI), tumor mutational burden (TMB), and tumor neoantigen burden (TNB) than well-differentiated neuroendocrine tumors (NET) (23, 29). Thus, they should be more immunogenic with an enhanced adaptive immune response and attract more lymphocyte infiltration, representing a preferred target for immunotherapy (37–39). However, lymphocytes infiltrated less in NEC than in ATC in this cohort, and NEC had a significantly lower PD-1 expression than ATC, which suggested that NEC in the thymus may not be that immunogenic and may not be responsive to immunotherapy as expected. Although previous literature in pulmonary NEN suggests that NEC may benefit more from ICB (23, 40), our clinical practice in a case with LCNEC (case No.3) showed resistance to ICB with toripalimab. To better define the intrinsic characteristics of NEN of the thymus, large-scale deep sequencing for MSI, TMB, and TNB in T-NEN is required in future studies.

Recent evidence has pointed out that PD-L1 expression should be best interpreted in the context of intratumoral T cell infiltration for the therapeutic prediction of ICB (17, 29, 40). From the perspective of PD-L1 expression and TILs, 39.2% (20/51) of T-NEN had type I cancer (PD-L1^+^TILs^+^) with a positive PD-L1 expression and the presence of T cell infiltration. They would potentially benefit from ICB according to the TILs/PD-L1 status classification of Teng et al (17). Five patients (5/51, 9.8%) appeared immunologically ignorant (PD-L1^-^TILs^-^). Therefore, single ICB probably would not have been successful, and combination treatment to enhance T cell infiltration would need to be considered in this category. Most T-NEN (26/51, 51%) in this study displayed a type IV landscape (PD-L1^-^TILs^+^) with the presence of TILs but the absence of PD-L1, which may still benefit from ICB according to previous literature (17). Although an association between the presence of PD-L1 and response to ICB has been reported, there are studies about patients with PD-L1-positive tumors who do not respond and patients with PD-L1-negative tumors who do respond (41). For instance, four NEN patients who showed PR in the Phase II KEYNOTE-158 study all displayed PD-L1 negative staining on their specimen (6). There are several reasons that may explain the observation of clinical response to ICB in certain cases of nominally PD-L1 negative tumors (42). First, tumor tissue acquired by needle biopsy may miss the PD-L1 positive area and thus provide false negative results. Second, tissue processing (freezing, formalin fixation) for IHC can alter epitopes and may potentially affect the PD-L1 staining. Third, PD-L1 expression is inducible and dynamic, its expression may vary from the time at which the biopsy is taken to the start of ICB treatment. Therefore, it is notable that a lack of detectable PD-L1 expression does not preclude anti-tumor activity in response to ICB. This point of view was confirmed by our case reports on two T-NEN patients (case No.1 and 2) with a type IV landscape (PD-L1^-^TILs^+^), who showed sustained PR after toripalimab treatment. It was reported that among multiple variables, the abundance of CD8^+^ TILs was the most predictive of the response to anti-PD-1/PD-L1 therapy across 21 cancer types (43). In this context, it is intriguing to note that type I and type IV immune landscapes, which were marked by the presence of TILs, predominated in T-NEN, making patients with T-NEN preferred candidates for immunotherapy trials.

Although this is a pioneer study, we acknowledge several limitations. First, this was a retrospective study based on two medical centers, which may have resulted in bias. Prospective evidence and multi-center studies are required to confirm these results. Second, our analysis was focused exclusively on primary tumors, thus lacking delineation of the immune landscape in metastatic lesions. Paired collection of samples from both primary and metastatic sites is necessary to compare their differences in terms of the immune landscape. Third, this study suffered from a limited sample size due to the rarity of T-NEN, and only a limited number of NEC were included in our study. Further validation of our findings in a larger cohort of patients is critical to minimize the biases possibly deriving from the heterogeneity. Fourth, due to the low incidence and relatively slow-growing nature of T-NEN, analyses of the prognostic relevance of the immune phenotype for overall survival is impracticable at this moment. Longer follow-up for subsequent survival status and prospective studies are still needed. Fifth, predictive biomarkers for ICB efficacy in T-NEN could not be identified given the limited cases received ICB treatment in this study cohort. A well-designed biomarker-guided clinical study is warranted to carry out in the future to identify reliable biomarker for predicting ICB efficacy in T-NEN.

## Conclusions

**Figure 5:**
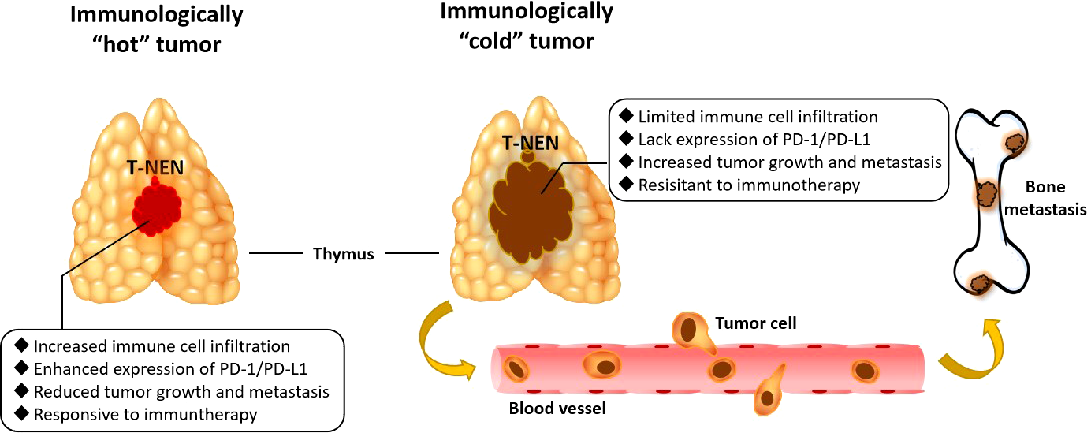
Graphical abstract of immunologically”hot”and”cold”tumors in T-NEN. Immunologically “hot” T-NEN with increased immune cell infiltration and enhanced expression of PD-1/PD-L1 tended to have restricted tumor size and less metastases, and would be more sensitive to immunotherapy. Immunologically “cold” T-NEN with limited immune cell infiltration and lacking expression of PD-1/PD-L1 tended to grow more aggressively and develop more metastases, especially to bones, and would be more resistant to immunotherapy.

Taken together, this was an innovative investigation of T-NEN, delineating for the first time the immune landscape, its association with clinical characteristics, and the potential response to immunotherapy in T-NEN. This comprehensive delineation showed us an overview that immunologically “hot” T-NEN with increased immune cell infiltration and enhanced expression of PD-1/PD-L1 tended to have restricted tumor size and less metastases. Therefore, they would be more sensitive to immunotherapy. On the other hand, immunologically “cold” T-NEN with limited immune cell infiltration and a lack of expression of PD-1/PD-L1 tended to grow more aggressively and develop more metastases, especially to bones, and would be more resistant to immunotherapy (**Figure 5**). This study also provided real-world clinical practice experience of immunotherapy with anti-PD-1 monoclonal antibody in patients with metastatic T-NEN. This may guide its future application by clinicians for this special group of patients. Collectively, our findings led to a more precise classification for T-NEN, and may enable more optimized and personalized clinical trials regarding immunotherapy for patients with T-NEN in the foreseeable future.

## Data availability statement

The original contributions presented in the study are included in the article/**Supplementary Material**. Further inquiries can be directed to the corresponding authors.

## Ethics statement

The studies involving human participants were reviewed and approved by Institutional Ethics Committee (IEC) for Clinical Research of the First Affiliated Hospital of Sun Yat-sen University. Written informed consent to participate in this study was provided by the participants’ legal guardian/next of kin.

## Author contributions

ML and JC designed the study. ML prepared the initial draft. ML and WH performed the experiment and data analysis. YZ collected the samples and assisted in the data collection. YL, NZ, YW, LC, and YJL provided technical support and guidance. JC and MC conceived the study and participated in the design of the study and contributed to the revision of the manuscript. All authors contributed to the article and approved the submitted version.

## Funding

The study was funded by the National Natural Science Foundation of China (grant number: 82141104), Natural Science Foundation of China (grant number: 82002502) and the Natural Science Foundation of Guangdong Province (grant number: 2019A1515012027).

## Conflict of interest

The authors declare that the research was conducted in the absence of any commercial or financial relationships that could be construed as a potential conflict of interest.

## Publisher’s note

All claims expressed in this article are solely those of the authors and do not necessarily represent those of their affiliated organizations, or those of the publisher, the editors and the reviewers. Any product that may be evaluated in this article, or claim that may be made by its manufacturer, is not guaranteed or endorsed by the publisher.

## Supplementary material

Supplementary Figure 1 Schematic of how immune cell density was assessed. Whole slides were scanned at ×20 magnification by the Axio Scan. Z1 Slide Scanner. Images were captured from five 1 mm^2^ areas on each slide. Quantification of positively stained cells was performed using ImagePro Plus software. The average number of cells of interest (number/mm^2^) was calculated based on the scores from the five 1 mm^2^ areas. The tumor area containing the highest density of associated markers was designated the “hotspot”.
