# Supplementary figures and images for: Clinical implications of immune checkpoint markers and immune infiltrates in patients with thymic neuroendocrine neoplasms
Source: Front Oncol. 2022 Sep 20;12:917743. doi: 10.3389/fonc.2022.917743 (PMC9531766; doi:10.3389/fonc.2022.917743)

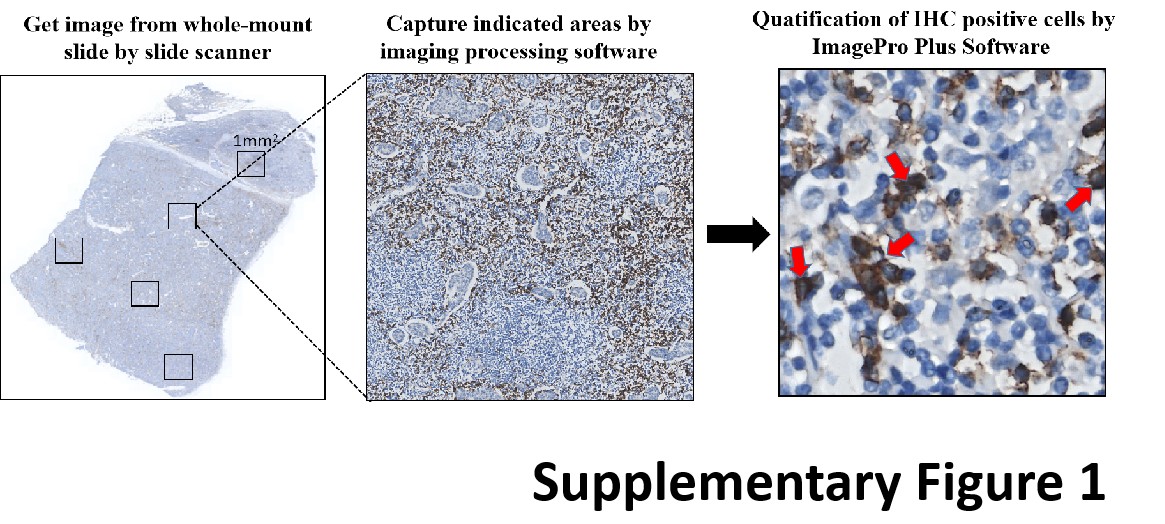

Supplement: Supplementary Figure 1 — Schematic of how immune cell density was assessed. Whole slides were scanned at ×20 magnification by the Axio Scan. Z1 Slide Scanner. Images were captured from five 1 mm2 areas on each slide. Quantification of positively stained cells was performed using ImagePro Plus software. The average number of cells of interest (number/mm2) was calculated based on the scores from the five 1 mm2 areas. The tumor area containing the highest density of associated markers was designated the “hotspot”. [file Image_1.jpeg]
