# Supplementary material for: Clinical implications of immune checkpoint markers and immune infiltrates in patients with thymic neuroendocrine neoplasms
Source: Front Oncol. 2022 Sep 20;12:917743. doi: 10.3389/fonc.2022.917743 (PMC9531766; doi:10.3389/fonc.2022.917743)
Supplement: Supplementary file 2 [file Table_1.docx]

**Supplementary Table 1. Information of irAE in the patients of T-NEN treated with ICB**

| **Category** | **Patients, No.** | |
| --- | --- | --- |
|  | **Grade 1-2** | **Grade 3-4** |
| **Skin** |  |  |
| -Rash | 0 | 0 |
| -Pruritus | 0 | 0 |
| -Vitiligo | 0 | 0 |
| **Pneumonitis** | 0 | 0 |
| **Myocarditis** | 0 | 0 |
| **Endocrine** |  |  |
| -Thyroiditis/hypothyroidism | 0 | 0 |
| -Hypophysitis | 0 | 0 |
| **Gastrointestinal** |  |  |
| -Mucositisf | 0 | 0 |
| -Diarrhea/colitisg | 0 | 0 |
| **Hepatobiliary** |  |  |
| -Hepatitis | 0 | 0 |
| -Cholangitis | 0 | 0 |
| **Renal dysfunction** | 0 | 0 |
| **Other** |  |  |
| -Fatigue | 1 | 0 |
| -Appetite loss | 1 | 0 |
| -Polyarthritis | 0 | 0 |
| -Myasthenia gravis | 0 | 0 |
